# T‐Cell Immunity and Lung Cancer

**DOI:** 10.1002/resp.70220

**Published:** 2026-02-11

**Authors:** Kotaro Yamada, Yosuke Togashi

**Affiliations:** ^1^ Department of Respiratory Medicine Okayama University Hospital Okayama Japan; ^2^ Department of Tumor Microenvironment Faculty of Medicine, Dentistry and Pharmaceutical Sciences, Okayama University Okayama Japan; ^3^ Faculty of Medicine, Kindai University Osaka Japan

**Keywords:** immune checkpoint inhibitors, lung neoplasms, T follicular helper cells, T‐cell exhaustion, T‐lymphocytes cytotoxic, T‐lymphocytes regulatory, tumour microenvironment

## Abstract

Lung cancer remains a leading cause of cancer mortality worldwide. Although immune checkpoint inhibitors (ICIs) have reshaped therapeutic strategies in lung cancer, their benefits remain limited. ICIs exert their therapeutic efficacy by activating T‐cell effector functions, underscoring the central role of T cells in antitumor immunity. Thus, this review focuses on the role of T cells in lung cancer and summarises recent advances. Tumour‐specific CD8^+^ T cells that attack tumour cells directly form the core of antitumor immunity, yet chronic antigenic stimulation drives functional impairment and exhaustion that constrain treatment responsiveness. Conversely, regulatory T cells modulate immune responses through diverse suppressive mechanisms and influence clinical outcomes. In addition, tertiary lymphoid structures (TLSs) that arise within tumours can amplify local immunity through interactions among follicular helper T cells, B cells, and other immune subsets, and are increasingly linked to therapeutic efficacy and prognosis. Emerging evidence also indicates that metabolic features of the tumour microenvironment modulate T‐cell differentiation and persistence. Collectively, these insights provide a foundation for translating an improved understanding of T‐cell–centred immune responses and their regulatory circuits into clinical practice. Overall, clarifying T‐cell functional states is essential for optimising immunotherapy and achieving durable benefit in lung cancer.

## 1 Introduction

As of 2022, lung cancer was the most commonly diagnosed malignancy worldwide and remained the leading cause of cancer‐related mortality, accounting for approximately 18.7% of all cancer deaths [1]. In 2015, the CheckMate 017 and 057 trials demonstrated that the anti–programmed cell death 1 (PD‐1) antibody, nivolumab, significantly prolonged overall survival (OS) compared with docetaxel in previously treated non–small cell lung cancer (NSCLC), thereby providing the first clinical evidence of the efficacy of immune checkpoint inhibitors (ICIs) in lung cancer [2, 3]. These pivotal studies marked a turning point in lung cancer therapy. Subsequently, novel treatment strategies have emerged, including combination regimens with anti–cytotoxic T‐lymphocyte–associated protein 4 (CTLA‐4) antibodies and chemoimmunotherapy approaches [4]. Based on first‐line ICI trials in advanced/metastatic NSCLC, the objective response rate (ORR) is generally 26%–65%, and the median OS is approximately 16–27 months [5]. Nevertheless, predicting therapeutic efficacy and expanding the population of responders remain major challenges.

ICIs exert their therapeutic efficacy by activating T‐cell effector functions, underscoring the central role of T cells in antitumor immunity. In fact, studies in mice have shown that ICIs are ineffective in the absence of T cells [6], and in patients with lung cancer, higher levels of T‐cell infiltration correlate with improved therapeutic efficacy [7]. In this review, we focus on the role of T cells in antitumor immunity, with particular emphasis on recent advances in the understanding of CD8^+^ T‐cell exhaustion and reinvigoration, the immunosuppressive functions of regulatory T (Treg) cells, the involvement of follicular helper T (Tfh) cells and tertiary lymphoid structure (TLS) formation, and metabolic dysfunction in T cells within the tumour microenvironment (TME).

## 2 Cancer Immunoediting and Cancer Immunotherapy

**FIGURE 1:**
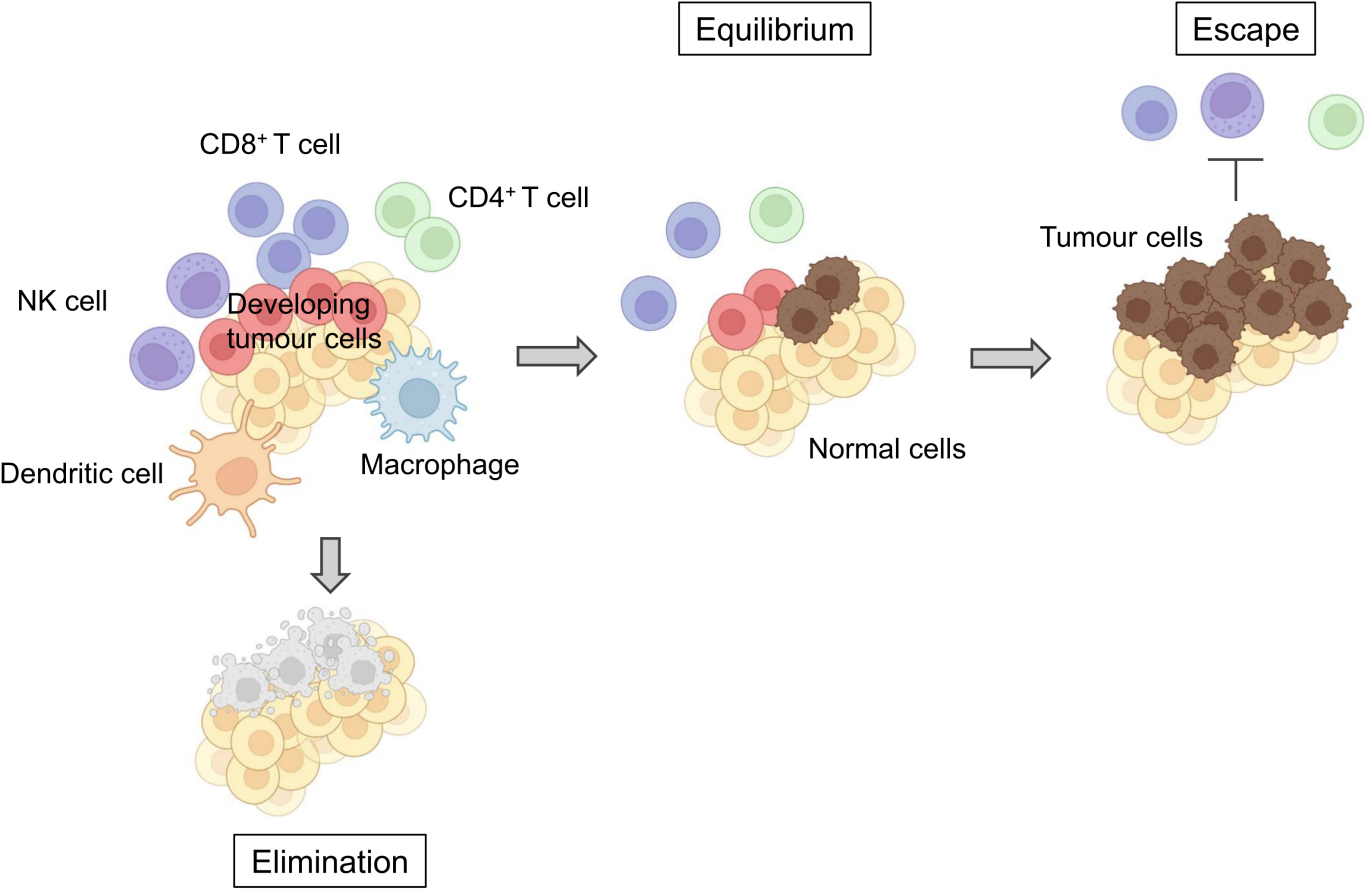
Cancer immunoediting. Cancer immunoediting describes the dynamic interaction between tumours and the immune system, comprising three phases: (1) Elimination—immune cells recognise and eliminate tumour cells; (2) Equilibrium—residual tumour cells persist in a dormant state under immune surveillance; and (3) Escape—immune‐resistant clones emerge and proliferate within an immunosuppressive microenvironment, leading to clinically detectable tumours. This figure was created based on Reference [9]. Created in https://BioRender.com.

It has been estimated that thousands of malignant cells arise daily in the human body; however, the immune system normally recognises these cells as non‐self and eliminates them, thereby preventing the development of clinically apparent cancer [8]—this stage is referred to as the “elimination phase.” In contrast, some tumour cells evade complete eradication and persist in a state of dormancy in which tumour progression is suppressed but not clinically detectable, known as the “equilibrium phase.” Over time, immune‐resistant clones may be selected, leading to the establishment of an immunosuppressive environment that allows tumour cells to escape immune surveillance and manifest as clinically detectable cancer—this is the “escape phase.” The concept of “cancer immunoediting” summarises the dynamic interaction between tumour cells and antitumor immunity across these three phases: elimination, equilibrium, and escape (Figure 1) [9, 10]. The present cancer immunotherapies including ICIs work to revert tumours in the escape phase back toward equilibrium—or ideally to the elimination phase—by reactivating immune surveillance. Among such immune surveillance systems, T cells are reported to play crucial roles.

## 3 The Seven Steps of T‐Cell Activation and Immune Checkpoint Molecules

**FIGURE 2:**
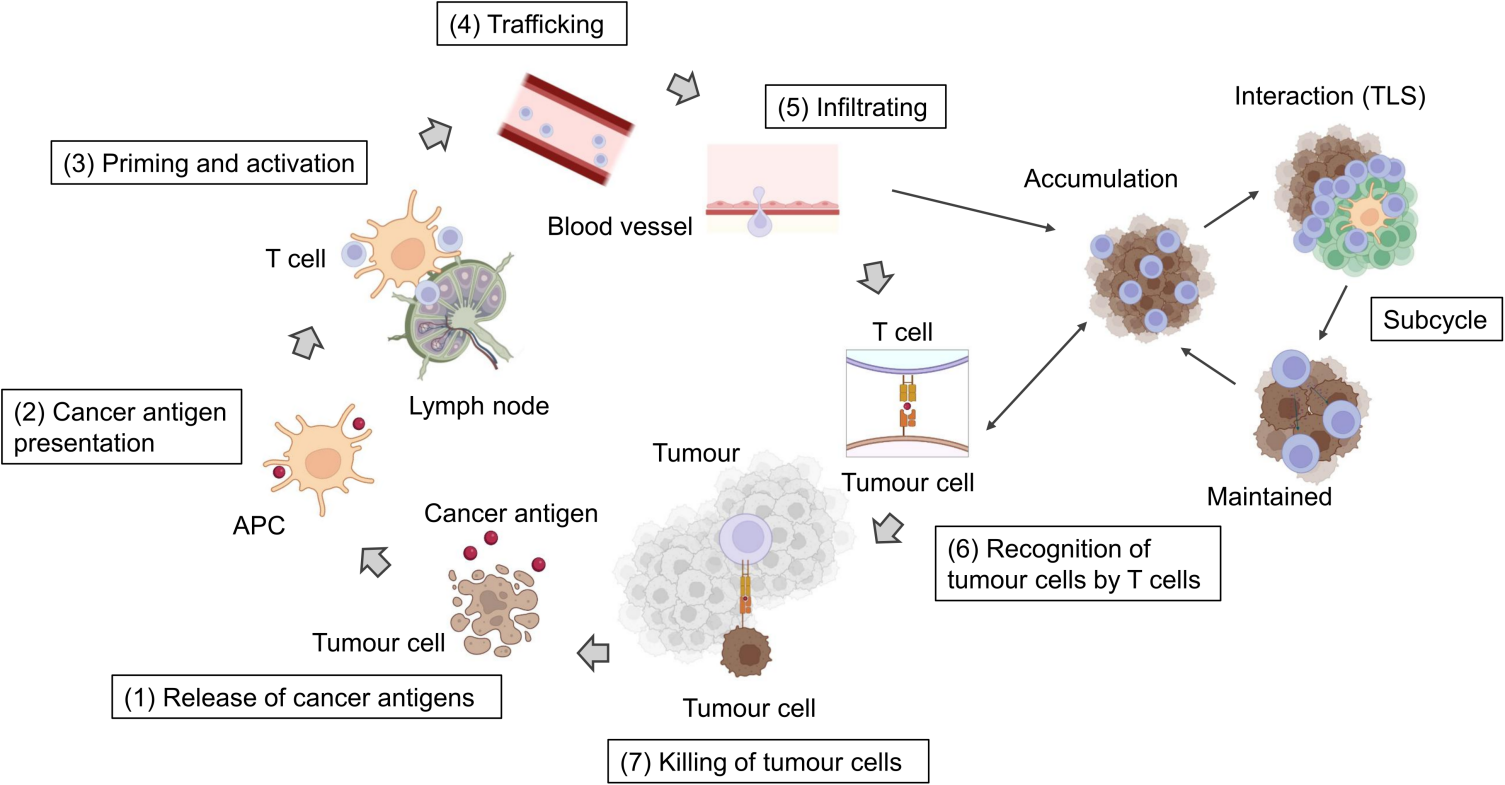
Cancer–immunity cycle with intratumoral subcycles. (1) Tumour cell death releases cancer antigens. (2) Antigen‐presenting cells (APCs), such as dendritic cells, capture and process these antigens. (3) In lymphoid tissues, APCs presenting cancer antigens via MHC prime naïve T cells and drive clonal expansion. (4) Activated T cells enter the circulation and home to tumours via chemokine cues and adhesion molecules. (5) Effector T cells extravasate and infiltrate the tumour parenchyma. (6) T‐cell receptors recognise peptide–MHC complexes on tumour cells, with co‐stimulatory and inhibitory checkpoints modulating the response. (7) Cytotoxic T cells kill tumour cells through perforin/granzyme and Fas–FasL pathways, releasing additional antigens and re‐engaging the cycle. The concept of a “subcycle” taking place within tertiary lymphoid structures (TLS) like draining lymph nodes that form adjacent to or within tumours has been proposed. TLS are composed of immune cells such as T cells, B cells, and dendritic cells. Through these structures, antitumor immunity can be locally amplified within the tumour microenvironment, where humoral and cellular immune responses act synergistically. This figure was created based on Reference [12]. Created in https://BioRender.com.

In 2013, the process of T‐cell activation was described as a “cancer–immunity cycle,” consisting of seven sequential steps [11]: (1) release of cancer antigens from tumour cells; (2) presentation of tumour antigens on major histocompatibility complex (MHC) molecules by antigen‐presenting cells (APCs); (3) recognition and priming of T cells in draining lymph nodes (priming phase); (4) trafficking of activated T cells; (5) infiltration into the TME; (6) recognition of cancer antigens presented on MHC molecules of tumour cells; and (7) killing of tumour cells (effector phase) (Figure 2).

During steps (3) and (6)/(7), T cells form an immunological synapse with APCs or tumour cells. At this interface, immune checkpoint molecules critically regulate the balance between T‐cell activation and inhibition [13]. Clinically available checkpoint inhibitors—anti–PD‐1/PD‐L1 and anti–CTLA‐4 antibodies—target these inhibitory molecules to release T cells from suppression. CTLA‐4 and PD‐1 suppress T‐cell activation through distinct mechanisms. Effective T‐cell activation requires not only signalling through the T‐cell receptor (TCR) but also co‐stimulatory signals, the prototypical example being CD28. CD28 expressed in T cells binds to CD80 and CD86 in APCs, delivering essential co‐stimulatory signals. CTLA‐4, however, has a higher affinity for CD80/CD86 than CD28, thereby sequestering these ligands and preventing CD28‐mediated co‐stimulation, which results in T‐cell inhibition [14]. Accordingly, anti–CTLA‐4 antibodies are thought to primarily act during the priming phase (step 3) of the cancer–immunity cycle. In contrast, PD‐1 is expressed in T cells and, upon binding to its ligands PD‐L1 or PD‐L2, delivers inhibitory signals that attenuate TCR‐mediated activation [15]. While PD‐1 was initially considered to act mainly during the effector phase (steps 6 and 7), high PD‐L1/PD‐L2 expression in APCs suggests that PD‐1 may also function during the priming phase [16]. Recent studies further indicate that PD‐1 can inhibit CD28‐mediated co‐stimulation in addition to TCR signalling [17, 18].

**FIGURE 3:**
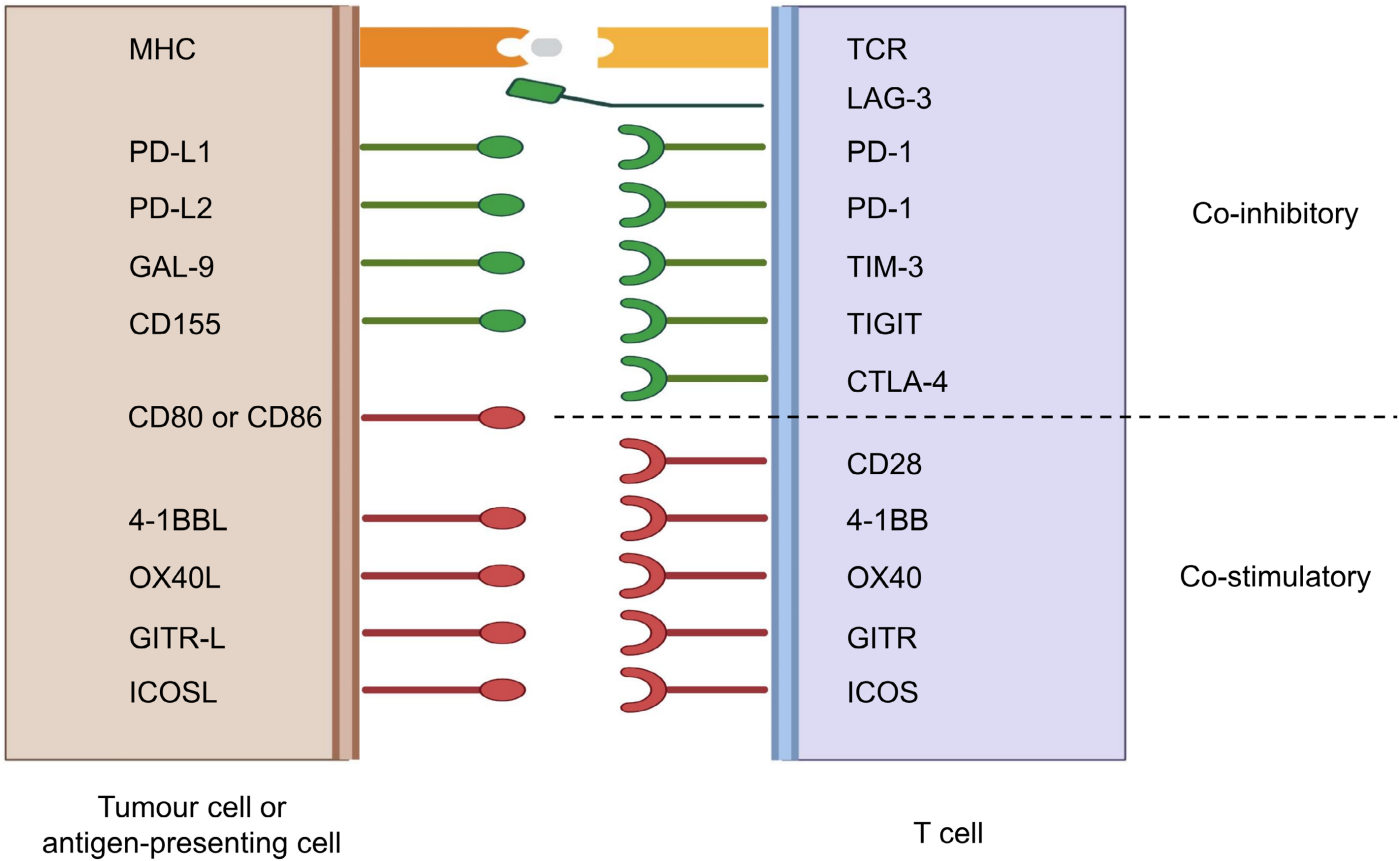
Representative immune checkpoint molecules at the immunological synapse. Interactions between tumour or antigen‐presenting cells (left) and T cells (right). Top, co‐inhibitory molecules including LAG‐3, PD‐1, TIM‐3, TIGIT, and CTLA‐4. Bottom, co‐stimulatory molecules such as CD28, 4‐1BB, OX40, GITR, and ICOS. This figure was created based on Reference [19]. Created in https://BioRender.com.

In addition to CTLA‐4 and PD‐1, numerous checkpoint molecules such as LAG‐3, TIM‐3, TIGIT, and ICOS have been identified. Some of these function as inhibitory receptors similar to CTLA‐4 and PD‐1 but operate through distinct molecular mechanisms. Conversely, certain co‐stimulatory checkpoint molecules provide activating signals that activate T‐cell function. Through their interplay, these molecules collectively contribute to the shaping of immune responses via diverse mechanisms (Figure 3) [13, 19].

## 4 Emerging Importance of TLS in the Cancer–Immunity Cycle

The priming phase (step 3) has traditionally been regarded as occurring within draining lymph nodes. However, in 2023, the concept of “subcycles” within the cancer–immunity cycle was proposed, highlighting the role of TLS that form within or adjacent to tumours (Figure 2) [12]. TLS arise in non‐lymphoid tissues under conditions of chronic inflammation, such as cancer or persistent infection, and are composed of abundant T cells, B cells, plasma cells, dendritic cells, and follicular dendritic cells [20]. In TLSs, dendritic cells (DCs) present antigens and activate CD4^+^ helper T cells, including Tfh cells, thereby initiating germinal centre reactions and B‐cell responses [12, 20]. Concurrently, T helper 1 (Th1) cells, via CD40L–CD40 signalling and interferon‐γ, drive DCs into a mature state, enhancing cross‐presentation and amplifying tumour‐specific CD8^+^ T‐cell priming [21]. Consequently, humoral and cellular immunity act synergistically within the TME [12, 20].

The most distinctive feature of TLSs is their localization within or adjacent to tumours, which confers several immunological advantages over secondary lymphoid organs such as lymph nodes or the spleen. These advantages include: (1) the ability to rapidly execute the process from priming to cytotoxicity, (2) the formation of localised immune niches that amplify immune responses, (3) the direct exposure of immune cells to the TME, which optimises immune activation, and (4) the potential to sustain effector T‐cell survival and possibly foster memory differentiation through repeated interactions with APCs and local survival factors within TLSs [20].

TLS formation is critically dependent on the chemokines CXCL13, CCL19, and CCL21, which recruit naïve T and B cells [20]. Among these, CXCL13 plays a particularly important role, as both Tfh cells and B cells express its receptor CXCR5, establishing a strong link between CXCL13 signalling and TLS development [20]. Tumour‐infiltrating PD‐1^+^ exhausted CD8^+^ T cells and Tfh cells, both of which possess antitumor activity, have been shown to produce CXCL13 and the abundance of these CXCL13‐producing subsets correlates with enhanced responsiveness to ICIs [22, 23]. Using single‐cell sequencing, Lowery et al. demonstrated that CXCL13‐producing exhausted CD8^+^ T cells and Tfh‐like CD4^+^ T cells in the TME had tumour‐cell reactivities [24]. In addition, combined with murine models, we demonstrated that tumour‐specific exhausted CD8^+^ T cells with antitumor reactivity highly express CXCL13, thereby promoting Tfh‐cell recruitment [25]. Furthermore, recent advances in single‐cell spatial analysis have further refined our understanding of TLS biology, revealing that exhausted CD8^+^ T cells localised within TLS differ from those positioned in direct proximity to tumour cells, and highlighting the importance of TLS‐associated T‐cell subsets in sustaining antitumor immunity (in submission).

Across solid tumours, the presence of mature TLSs—which are enriched for tumour‐specific T and B cells—has been associated with enhanced efficacy of anti–PD‐1/PD‐L1 therapy independently of tumour PD‐L1 expression [26]. In NSCLC, TLS abundance and maturation are independently associated with longer relapse‐free survival and OS after multivariable adjustment [20, 27]. Evidence from malignancies typically refractory to ICIs further shows that responses are enriched among TLS‐positive cases, supporting TLS‐based stratification as a promising predictive biomarker [28]. In resected specimens obtained after neoadjuvant chemoimmunotherapy, increases in TLS number and maturation have been observed, consistent with a role for TLSs as a local niche that augments ICI responsiveness [29]. Notably, TLS frequency and density appear to vary by stage: in treatment‐naïve surgical cases (stage I–III), TLSs are detected at high rates (67%–90%), whereas in unresectable stage III–IV disease before ICI initiation, prevalence is approximately 30% [30, 31]. In addition, among patients with post‐resection recurrent NSCLC, extensive lymphadenectomy has been associated with diminished benefit from immunotherapy [32]. Taken together with evidence that TLSs can assume secondary lymphoid organ–like functions at the tumour site, these observations support TLS‐informed selection of ICI‐containing regimens as an immunologically rational strategy.

## 5 Effector Functions and Exhaustion of CD8 ^+^ T Cells

T cells are broadly classified into CD4^+^ and CD8^+^ subsets, each with distinct functions (Table 1). CD4^+^ T cells mainly act as helper cells, orchestrating and regulating immune responses through cytokine production, whereas CD8^+^ T cells function as cytotoxic lymphocytes that directly eliminate target cells [21]. In the context of tumour immunity, CD8^+^ T cells recognise tumour antigens presented on MHC class I molecules on the surface of tumour cells and exert cytotoxic activity against these targets [12]. Upon activation through TCR signalling in combination with co‐stimulatory signals such as CD28, CD8^+^ T cells exert cytotoxic functions by expressing perforin, granzymes, and Fas ligand, thereby inducing tumour cell killing [33, 34]. In murine models, depletion of CD8^+^ T cells results in accelerated tumour growth and abrogates the efficacy of ICIs [6]. Thus, CD8^+^ T cells play a central role in cancer immunotherapy.

**TABLE 1:** Summary of T‐cell subsets discussed in this review.

	Subsets	Phenotypic markers/Transcription factors	Functions
CD8^+^ T cell	Effector	T‐bet^+^, CD44^+^, CD62L^−^, KLRG1^+^	Cytotoxic killing via GZMB/PRF1 and IFN‐γ–mediated antitumor inflammation.
	Exhausted		Tumour‐reactive T cells that acquire dysfunction under chronic antigen stimulation, forming hierarchical exhaustion including progenitor and terminal subsets.
	Progenitor exhausted	TCF1^+^, PD‐1^int^, TIM‐3^−^, LAG‐3^−^, CXCR5^+^	Stem‐like exhausted subset that sustains the exhausted pool and is highly responsive to PD‐1 blockade.
	Terminally exhausted	TCF1^−^, PD‐1^hi^, TIM‐3^+^, LAG‐3^+^, CXCR5^−^	Dysfunctional, cytokine‐poor state that contributes to immune evasion and responds poorly to ICI.
CD4^+^ T cell	Th1	T‐bet^+^, CXCR3^+^, CCR5^+^	Produce IFN‐γ to activate DCs and support CD8 priming, driving type‐1 antitumor immunity.
	Th2	GATA3^+^, CCR4^+^	Produce IL‐4/5/13 and promote type‐2 inflammation and an immunosuppressive TME.
	Th17	RORɤt^+^, CCR6^+^	Produce IL‐17 and induce inflammation/angiogenesis; effects are context‐dependent in tumours.
	Treg	Foxp3^+^, CD25^+^, CTLA‐4^+^	Suppress effector T cells and DCs, shaping an immunosuppressive TME; often linked to poor prognosis.
	Tfh	BCL6^+^, CXCR5^+^, PD‐1^+^, ICOS^+^	Promote B‐cell help and CXCL13‐dependent TLS formation, enhancing antitumor immunity and ICI response.

Abbreviations: DCs, dendritic cells; GZMB, granzyme B; ICI, immune checkpoint inhibitor; PRF1, perforin 1; TLS, tertiary lymphoid structure; TME, tumour microenvironment.

Under conditions of persistent antigen exposure, however, T cells may enter a dysfunctional state known as “exhaustion.” Originally described in the context of chronic viral infection [35], this phenomenon is likewise widely observed within tumours, where tumour‐infiltrating CD8^+^ T cells exhibit an exhausted phenotype characterised by the co‐expression of inhibitory receptors, accompanied by reduced proliferative capacity and diminished cytokine production [36, 37]. Immune checkpoint molecules such as PD‐1 play a pivotal role in mediating this dysfunctional state. Chronic antigenic stimulation induces high PD‐1 expression on T cells; continuous engagement with PD‐L1 delivers inhibitory signals that drive functional exhaustion [38, 39].

Recent analyses have revealed that tumour‐infiltrating CD8^+^ T cells consist of heterogeneous populations, including tumour‐reactive clones and bystander clones without direct antitumor activity. Notably, PD‐1^−^CD8^+^ T cells tend to reside within the stromal compartment, whereas PD‐1^+^ CD8^+^ T cells are localised near tumour cells, suggesting that PD‐1^+^ tumour‐infiltrating CD8^+^ T cells represent the clonotypes actively engaged in tumour cell recognition and cytotoxicity [22, 40]. Using single‐cell transcriptomics coupled with TCR sequencing, we and others have identified expanding CD8^+^ T‐cell clones that express PD‐1 and display an exhausted phenotype. Such exhausted T‐cell clones exhibited reactivity against autologous tumour cells, whereas non‐exhausted T‐cell clones were abundant in the peripheral blood yet demonstrated no tumour reactivity even with clonal expansion. These findings support the concept that ICIs reinvigorate tumour‐specific, PD‐1^+^ exhausted CD8^+^ T cells, thereby restoring antitumor immunity [41, 42, 43], and further suggest that the abundance of intratumoral PD‐1^+^CD8^+^ T cells may serve as a promising biomarker for predicting the therapeutic efficacy of ICIs [22, 44].

Furthermore, distinct differentiation states within exhausted T cells have been implicated in therapeutic responses and resistance. PD‐1 blockade monotherapies appear to preferentially act on progenitor exhausted CD8^+^ T cells, which express TCF‐1 and CXCR5 with intermediate levels of PD‐1, restoring their proliferative capacity, cytokine secretion, and intratumoral migration. By contrast, terminally exhausted CD8^+^ T cells, characterised by high PD‐1 expression with reduced TCF‐1 and CXCR5 expression, fail to respond to PD‐1 blockade monotherapies, and the expansion of this population is considered a major mechanism of therapeutic resistance [45, 46]. Indeed, terminally exhausted T cells often co‐express additional inhibitory receptors such as LAG‐3, TIM‐3, and TIGIT, rendering PD‐1 or CTLA‐4 blockade insufficient for functional restoration [47, 48, 49]. Consistent with this, we have demonstrated in clinical specimens that TIGIT and its ligand CD155 contribute to resistance to PD‐1 and CTLA‐4 blockade, and experimental blockade of this pathway effectively reverses resistance [50].

In NSCLC, as in other malignancies, exhausted CD8^+^ T cells have been reported to play an important role in antitumor immunity, and their characteristic subsets are attracting attention as candidate indicators for predicting therapeutic efficacy and prognosis [51, 52]. For example, exhausted CD8^+^ T cells expressing CD39 and/or CD103 have been suggested to be associated with responsiveness to ICIs and with favourable recurrence‐free or overall outcomes [51, 53]. In addition, progenitor exhausted CD8^+^ T cells have been reported to localise preferentially near TLSs in lung cancer, and their significance as a response‐competent pool has been discussed [52]. The differentiation state of exhaustion and the spatial context of these cells may serve as potential predictive markers of treatment response and prognosis.

## 6 CD4 ^+^ T Cells: Conventional Helper Subsets (Th1, Th2, Th17)

The role of CD4^+^ T cells in tumour immunity remains a subject of debate, and studies in murine tumour models have shown that depletion of CD4^+^ cells can enhance antitumor responses [54]. Such findings suggest that CD4^+^ T cells may exert heterogeneous effects depending on the immunologic context.

Conventional helper T‐cell subsets—Th1, T helper 2 (Th2), and T helper 17 (Th17)—are known to contribute to antitumor immunity in diverse ways [21, 55]. Th1 cells, defined by T‐bet expression and IFN‐γ production, promote dendritic‐cell maturation and support the priming of tumour‐specific CD8^+^ T cells, and Th1‐oriented immune profiles are generally associated with improved tumour control and better responses to ICIs [21, 55]. In contrast, Th2 cells, characterised by GATA3 and IL‐4/IL‐5/IL‐13 secretion, promote alternatively activated macrophages and suppress type 1 inflammation, thereby contributing to an immunosuppressive TME [21]. Th17 cells, driven by RORγt and producing IL‐17A, exhibit context‐dependent effects: IL‐17–mediated inflammation and stromal remodelling may facilitate tumour progression, although Th17 responses can also support early antitumor immunity under specific conditions [21, 55]. Overall, conventional helper T cells modulate the cytokine milieu and co‐stimulatory landscape, thereby influencing the activation and functional state of CD8^+^ T cells within the TME.

## 7 CD4 ^+^ T Cells: Treg Cells

In contrast to CD8^+^ T cells, which mediate antitumor immune responses, Treg cells, characterised by the expression of Foxp3 [56], are known to suppress antitumor immunity. Foxp3 functions as a master transcription factor governing the differentiation, stability, and suppressive program of Treg cells, integrating diverse immunoregulatory pathways into a coherent transcriptional network [56, 57]. In advanced NSCLC, a higher intratumoral Foxp3^+^/CD8^+^ ratio assessed on pretreatment tumour biopsies independently predicts poor response to platinum‐based chemotherapy [58]. Moreover, in NSCLC, the intratumoral abundance and activation state of Treg cells are implicated in clinical outcome, with activated Treg‐cell signatures associated with resistance to PD‐1 blockade [59, 60]. We also previously reported that epidermal growth factor receptor (EGFR) activation regulates chemokine production, creating an immunosuppressive TME in *EGFR*‐mutated NSCLC, characterised by reduced CD8^+^ T‐cell infiltration and increased Treg‐cell infiltration [61]. Accordingly, *EGFR*‐mutated NSCLCs are characterised by poor responses to ICIs [62].

Treg cells employ multiple immunosuppressive mechanisms. One key mechanism involves the consumption of IL‐2. Foxp3 represses IL‐2 gene transcription, and thus Treg cells themselves are unable to produce IL‐2 [63]. However, they constitutively express high levels of the IL‐2 receptor α‐chain (CD25), which allows them to preferentially consume IL‐2 required for the proliferation of effector T cells, thereby indirectly suppressing the expansion of other T‐cell subsets [64, 65]. Another major mechanism is mediated by CTLA‐4, which is highly expressed in Treg cells. CTLA‐4 has a stronger affinity for CD80 and CD86 than CD28, thereby sequestering these ligands and preventing CD28‐mediated co‐stimulation. In addition, through trogocytosis, CTLA‐4 physically removes CD80/CD86 from the APC surface, further impairing co‐stimulation and ultimately reinforcing immunosuppression [66, 67]. Beyond IL‐2 consumption and CTLA‐4–mediated inhibition, Treg cells suppress immune responses through multiple effector functions. These include the secretion of immunosuppressive cytokines such as IL‐10, IL‐35, and TGF‐β; the release of cytotoxic mediators such as granzymes and perforin; and the generation of extracellular adenosine via the ectoenzymes CD39 and CD73 [68].

The role of Treg cells in resistance to PD‐1/PD‐L1 blockade has also been intensively investigated. Using flow cytometry and multiplex immunohistochemistry, we demonstrated that PD‐1 blockade can paradoxically activate PD‐1^+^ Treg cells, thereby suppressing antitumor immunity and leading to resistance or even rapid disease progression in certain patients including lung cancer ones [44, 69]. Similarly, by just blocking CTLA‐4, anti–CTLA‐4 antibodies liberate CD80/CD86, which can then provide co‐stimulatory signals to Treg cells via CD28, potentially augmenting CTLA‐4–independent suppressive functions. Therefore, the antitumor efficacy of anti–CTLA‐4 antibodies is thought to rely heavily on their ability to deplete Treg cells through antibody‐dependent cellular cytotoxicity (ADCC) [70].

## 8 CD4 ^+^ T Cells: Tfh Cells

Tfh cells are a subset of CD4^+^ T cells that play a central role in controlling B cell responses within germinal centres and B cell follicles. Tfh cells characteristically express the transcription factor Bcl‐6 and surface markers such as CXCR5, ICOS, and PD‐1. In addition, they produce the cytokine IL‐21 and IL‐4, which provide essential signals for B cell activation, class switching, and differentiation into plasma cells and memory B cells [71]. The presence of Tfh cells has also been confirmed in the TME. In particular, within TLSs formed at tumour sites, Tfh cells interact with B cells to activate antitumor immune responses. Tfh cells in TLSs not only induce B cells to produce tumour antigen–specific antibodies, but also support B cells in functioning as APCs, thereby promoting the activation of tumour antigen–specific T cells. B cells internalise tumour antigens and present them to CD4^+^ T cells through MHC class II, thereby assisting their activation, while simultaneously forming a positive feedback loop with Tfh cells through CD40–CD40L interactions and cytokines (particularly IL‐21 and IL‐4) [71]. Moreover, IL‐21 and IL‐4 derived from Tfh cells also contribute to the differentiation and functional maintenance of CD8^+^ T cells [72, 73, 74], thereby improving the quality of cytotoxic T cell responses at tumour sites. Indeed, in cases with intratumoral TLSs, B cells, Tfh cells, and CD8^+^ T cells are histologically located in close proximity and act cooperatively to strengthen antitumor immunity [75]. We reported that the antitumor effect of anti–PD‐1 antibodies was attenuated in Tfh cell–knockout mice and further demonstrated that Tfh‐like exhausted CD4^+^ T cells can exert direct cytotoxicity against tumour cells expressing MHC class II [25].

In lung cancer as well, tumour‐infiltrating Tfh cells have been suggested to contribute to favourable outcomes [76, 77]. Using mouse models, we also showed that combined treatment with anti–PD‐1 and anti–CTLA‐4 antibodies elicited antitumor effects against intracranial tumours, initiated by the activation of Tfh cells highly expressing CTLA‐4. In Tfh‐cell knockout mice, however, little enhancement of antitumor effects was observed, suggesting that Tfh cells play an important role in antitumor immunity [78]. In line with this preclinical Tfh dependence, human NSCLC brain metastases show TLS‐like aggregates composed of Tfh and B cells, implying a Tfh‐centred local immune circuit that may support therapeutic efficacy in the brain [78]. Consistently, a Japanese multicentre phase II trial showed that first‐line nivolumab plus ipilimumab with short course platinum chemotherapy achieved an intracranial ORR of 50% (including 20% complete responses) in patients with untreated brain metastases from NSCLC, supporting the clinical relevance of brain‐resident immune circuits [79].

## 9 T‐Cell Metabolism and Mitochondrial Dysfunction

The TME of lung cancer is characterised by nutrient deprivation, hypoxia, acidosis, altered lipid metabolism, and the accumulation of adenosine, all of which collectively impair T‐cell function [80]. Tumour cell consumption of glucose and tryptophan drives T‐cell dysfunction [81, 82], while the accumulation of lactate and adenosine inhibits the cytotoxic activity of CD8^+^ T cells and simultaneously enhances the immunosuppressive functions of Treg cells [83, 84, 85, 86]. Furthermore, hypoxia promotes the stabilisation of Treg cells via hypoxia‐inducible factor‐1α, underscoring the strong link between tumour metabolism and immune evasion [87].

We recently identified a novel mechanism of immune escape involving the horizontal transfer of mitochondria from tumour cells to T cells. Using clinical samples from patients with melanoma and NSCLC, we discovered that tumour‐infiltrating CD8^+^ T cells and tumour cells shared identical mitochondrial DNA (mtDNA) mutations, indicating direct mitochondrial transfer from tumour cells to T cells. Mechanistically, this transfer occurred via tunnelling nanotubes and exosomes [88, 89]. Acquisition of mutant mitochondria suppressed oxidative phosphorylation, leading to increased reliance on glycolysis, cellular senescence, and impaired memory formation and activation of T cells. These metabolic derangements contributed to reduced responsiveness to ICIs, and the presence of mtDNA mutations in tumours was suggested to be a negative prognostic factor for ICI therapy [88].

## 10 Predictive T‐Cell Biomarkers for ICI Response

The efficacy of ICIs depends largely on the quality of T‐cell responses within the TME. Although PD‐L1 expression, tumour mutational burden, and driver alterations (e.g., EGFR, ALK) influence treatment outcomes, these tumour‐intrinsic factors do not directly reflect T‐cell competence [90, 91, 92]. This section summarises T cell–related biomarkers that more accurately capture determinants of ICI responsiveness in lung cancer [90, 91, 93].

### 10.1 Tumour‐Reactive/Exhausted CD8 ^+^ T Cells

The overall density of CD8^+^ T cells does not adequately predict ICI responsiveness; instead, the quality of subsets enriched for tumour‐reactive clonotypes is more informative. CD8^+^ T cells with high PD‐1 expression, CD39^+^ CD8^+^ T cells, and CD39^+^CD103^+^ tissue‐resident memory–like cells delineate tumour‐specific populations whose abundance correlates with greater reinvigoration following ICI therapy [22, 51, 53]. In addition, progenitor exhausted T cells serve as a precursor pool that supports clonal expansion after treatment, and the hierarchical organisation within the exhausted CD8^+^ T‐cell compartment itself represents an important biomarker of therapeutic responsiveness [45, 46, 52].

### 10.2 TLS And CXCL13‐Producing T Cells

The presence of mature TLS is associated with improved ICI responses independent of PD‐L1 expression [26, 28]. TLS development is promoted by CXCL13‐producing Tfh‐like CD4^+^ T cells and exhausted CD8^+^ T cells, which recruit and organise lymphocytes within the tumour [23, 24, 25]. Thus, TLS abundance and maturity, together with CXCL13‐related signatures, serve as indicators of a TME capable of supporting effective T‐cell activation [26, 27, 29, 31].

### 10.3 Treg‐Mediated Immunosuppression

An increased intratumoral Treg population and a higher Foxp3^+^/CD8^+^ ratio reflect an immunosuppressive TME and correlate with reduced responsiveness to ICIs [58, 59, 60]. Of note, PD‐1^+^ Tregs may become further activated following PD‐1 blockade, underscoring that the balance between regulatory and effector T cells is a critical determinant of therapeutic outcome [44, 69].

## 11 Development of Novel Therapeutic Strategies

While the introduction of ICIs has driven substantial advances in lung cancer therapy, limitations in response rates and the development of acquired resistance remain incompletely resolved. To achieve greater therapeutic benefit, treatment design must be explicitly oriented toward the activation of T‐cell responses. In this section, we summarise the principal approaches currently under investigation.

### 11.1 Reinvigorating Exhausted CD8 ^+^ T Cells: Combination of Emerging Immune Checkpoints

In TME with chronic antigen exposure, CD8^+^ T cells upregulate inhibitory immune checkpoint receptors such as PD‐1 and transition to an exhausted state characterised by diminished IFN‐γ and granzyme production, reduced proliferative capacity, and increased susceptibility to apoptosis [37]. Beyond PD‐1, expression of various inhibitory immune checkpoint receptors including TIGIT, LAG‐3, and TIM‐3 also increased so‐called terminal exhaustion [36]. Because these receptors act at distinct nodes, their inhibitory circuits operate in parallel, allowing residual suppression to persist after PD‐1 blockade alone. Accordingly, combination strategies that concurrently inhibit residual inhibitory signals by targeting TIGIT, LAG‐3, TIM‐3, and related receptors are under active investigation. Clinically, dual LAG‐3/PD‐1 blockade improved progression‐free survival and gained regulatory approval in melanoma [94], whereas in NSCLC it remains under evaluation in neoadjuvant and advanced‐disease settings [95]. By contrast, in first‐line NSCLC with high PD‐L1 expression, a phase III TIGIT program did not demonstrate a statistically significant OS benefit [96], and an add‐on approach in first‐line small‐cell lung cancer (SCLC) likewise failed to meet its primary endpoints [97]. For TIM‐3, early‐phase studies have established safety and pharmacodynamic activity, but clinical efficacy in lung cancer has not yet been demonstrated [98]. Collectively, these observations underscore the heterogeneity of the exhaustion program and the need to align targets with tumour type, disease stage, and line of therapy.

Moreover, bispecific antibodies (BsAbs) enable dual pathway blockade within a single molecule, aligning the exposures of both activities under a shared pharmacokinetic profile to reduce dose/exposure mismatches inherent to separate‐drug combinations while aiming for additive or synergistic efficacy and optimised toxicity [99]. For LAG‐3, beyond simple co‐administration, PD‐1 × LAG‐3 BsAbs are being investigated, with ongoing trials in NSCLC across first‐line and previously treated settings [100]. In parallel, PD‐1 × CTLA‐4 BsAbs—notably cadonilimab, which has shown efficacy and received regulatory approval in cervical cancer [101]—are also under clinical evaluation in NSCLC [102].

### 11.2 Co‐Stimulatory Agonists

T‐cell activation requires co‐stimulation in addition to TCR signalling. Although CD28, a prototypic co‐stimulatory receptor, belongs to the immunoglobulin superfamily, this section focuses on co‐stimulatory receptors in the tumour necrosis factor receptor superfamily (TNFRSF), namely OX40 (CD134), 4‐1BB (CD137), CD27, and GITR [34]. As noted in the previous section, exhausted T cells express multiple inhibitory pathways in parallel, and therefore ICI therapy alone may not fully restore their function. To overcome this limitation, co‐stimulatory agonists, which deliver positive activation signals to reinvigorate exhausted T cells, have gained increasing attention. Agonist antibodies to these TNFRSF receptors potentiate TRAF‐dependent NF‐κB, MAPK, and PI3K–AKT signalling, thereby augmenting T‐cell proliferation, survival, cytokine production, and memory differentiation [103, 104, 105, 106].

Among these receptors, 4‐1BB agonists possess strong co‐stimulatory activity but have been limited by hepatotoxicity; 4‐1BB stimulation has been shown to activate hepatic myeloid cells and induce IL‐27–dependent hepatitis [107]. This challenge has prompted the development of strategies that restrict 4‐1BB activation to the TME through conditional co‐stimulation. The PD‐L1 × 4‐1BB bispecific antibody acasunlimab exemplifies this approach and has demonstrated reinvigoration of exhausted T cells and antitumor activity in preclinical models [108, 109], along with improved safety and emerging clinical activity in early trials for metastatic NSCLC [110]. Collectively, these findings indicate that co‐stimulatory agonists may complement ICI therapy and more effectively restore exhausted T‐cell function as a next‐generation immunotherapeutic strategy.

### 11.3 Selective Targeting of Intratumoral Treg Cells

Within the TME, dominance of Treg cells has been linked to primary and acquired resistance to PD‐(L)1 blockade. Accordingly, drug‐development efforts aim to selectively deplete or attenuate intratumoral Treg cells while preserving peripheral immune homeostasis. One approach involves CCR8‐targeting antibodies, which exploit the enrichment of CCR8 on human intratumoral effector Treg cells and seek to achieve local Treg‐cell control via ADCC/antibody‐dependent cellular phagocytosis mechanisms [111]. By contrast, CCR4‐directed strategies, although evaluated earlier in solid tumours, have shown limited efficacy and have not demonstrated a clear OS benefit in lung cancer [111]. In parallel, Fc‐engineered anti–CTLA‐4 antibodies with enhanced Fcγ receptor binding and Treg‐cell depleting capacity are being redesigned to address the therapeutic window and Treg‐cell selectivity limitations of conventional anti–CTLA‐4; clinical testing, including combinations with PD‐(L)1 inhibitors, is ongoing [112]. Conceptually, strategies grounded in tumour selectivity (differential target expression) and effector engagement (optimised Fc biology) aim to preferentially restrain Treg cells within the TME while restoring antitumor effector T‐cell activity.

### 11.4 Vascular Endothelial Growth Factor (VEGF) Inhibition

Vascular Endothelial Growth Factor (VEGF) promotes an immunosuppressive tumour milieu by sustaining immature vasculature, hypoxia, and reduced adhesion molecule expression, thereby impairing antigen presentation and hindering T‐cell infiltration [113]. Pharmacologic VEGF blockade can induce transient vascular normalisation, improving dendritic cell function and intratumoral T‐cell trafficking, which in turn facilitates the clinical activity of ICIs. Preclinically, combined anti‐VEGF and anti–PD‐L1 therapy has been shown to induce high endothelial venules and enhance cytotoxic T‐lymphocyte infiltration [114]. Clinically, first‐line atezolizumab plus bevacizumab and chemotherapy improved overall and progression‐free survival, supporting the efficacy of this immuno‐angiogenic approach [115]. Moreover, the PD‐1 × VEGF bispecific antibody ivonescimab has demonstrated progression‐free survival benefit over pembrolizumab in a phase III trial in PD‐L1–positive NSCLC [113]. By contrast, lenvatinib, a multikinase tyrosine kinase inhibitor targeting VEGFR/Fibroblast Growth Factor Receptor among others, has not shown consistent incremental benefit in phase III trials of ICI combinations in NSCLC [116]. Overall, VEGF inhibition represents a design principle that can bolster T‐cell immunity through multiple mechanisms, and in current clinical practice combination strategies exemplified by bevacizumab‐based regimens and PD‐1 × VEGF bispecific antibodies are becoming established.

### 11.5 Induction and Functional Enhancement of TLS

TLSs support local T‐cell priming within the TME and are associated with improved clinical outcomes under immune checkpoint blockade [20]. Building on mechanistic insights into TLS biology and antigen presentation, CD40 agonists and STING agonists are being evaluated to activate dendritic cells and strengthen cross‐priming, thereby potentially augmenting TLS function and downstream antitumor T‐cell responses [20, 117]. In early clinical studies, CD40 agonists have consistently produced pharmacodynamic evidence of myeloid and T‐cell activation in patients, yet antitumor activity has generally been modest outside of selected settings [118, 119]. By contrast, several intratumoral or systemic STING agonists have demonstrated target engagement but limited clinical efficacy as monotherapy and mixed results in combinations, prompting continued optimization of molecular design, route of administration including intratumoral delivery, and combinatorial partners [120, 121].

### 11.6 Redesigned Cytokine Therapy

Cytokines provide the third signal for T‐cell activation beyond TCR engagement and co‐stimulation, governing proliferation, survival, and differentiation. Among common γ‐chain cytokines (IL‐2, IL‐7, IL‐15, IL‐21), conventional high‐dose IL‐2 is constrained by preferential Treg‐cell expansion and systemic toxicities. These limitations have driven engineering efforts focused on receptor selectivity (βγ‐biased IL‐2 variants), tumour localization (immunocytokines and local delivery), and persistence optimization [122]. βγ‐selective IL‐2 variants such as nemvaleukin and pegenzileukin are being developed to preferentially activate CD8^+^ T cells and NK cells [123, 124]. In contrast, the PEGylated IL‐2 prodrug bempegaldesleukin (NKTR‐214) failed to demonstrate efficacy in phase III testing [125, 126]. IL‐15 super agonists (e.g., N‐803) have shown pharmacodynamic activity and acceptable safety in combination with anti–PD‐1 in metastatic NSCLC [127]. Tumour‐targeted immunocytokines (e.g., FAP‐IL2v) are intended to concentrate IL‐2 activity within the tumour, with early clinical signals reported [128]. Overall, receptor selectivity, tumour localization, and optimised combinations constitute the prevailing design principles for contemporary cytokine therapy [122].

### 11.7 Metabolic Reprogramming of T Cells

The mechanisms by which metabolic stress in the TME impairs T‐cell bioenergetics and function were summarised in the preceding section. This section focuses on therapeutic interventions that alleviate or reprogram these constraints. In the adenosine axis, development includes A2A/A2B receptor antagonists as well as inhibition of CD73/CD39 to reduce extracellular adenosine generation. Clinically, A2A antagonists have shown pharmacodynamic activity and acceptable tolerability in combination with anti–PD‐(L)1 therapy [129]. In NSCLC, combinations that include anti‐CD73 antibodies in the post‐definitive chemoradiotherapy consolidation setting have yielded phase II signals and have proceeded to phase III evaluation [130, 131]. Targeting the lactate‐rich and acidic TME seeks to normalise pH and metabolic load in order to preserve cytokine production and survival of cytotoxic T lymphocytes, using inhibitors of monocarboxylate transporters (MCTs) or carbonic anhydrase IX (CAIX). These approaches are in early clinical development [132, 133]. In amino‐acid metabolism, inhibition of indoleamine 2,3‐dioxygenase 1 (IDO1), which addresses tryptophan depletion and kynurenine accumulation, failed to meet primary endpoints in a large phase III trial, indicating a need for further refinement of target selection and combination design [134]. For lipid and mitochondrial metabolism, strategies such as AMP‐activated protein kinase (AMPK) activation (e.g., metformin) and restoration of mitochondrial function aim to support T‐cell persistence and memory. Randomised trials in NSCLC that combined metformin with chemoradiotherapy have not demonstrated benefit. Patient selection informed by molecular context, including LKB1/STK11 alterations, and optimization of combinations and dosing with PD‐(L)1 blockade remain active areas of investigation [135, 136, 137, 138].

### 11.8 Bispecific T‐Cell Engagers

T‐cell engagers (TCEs), including bispecific T‐cell engagers, are antibodies that simultaneously bind CD3 on T cells and a tumour‐associated antigen, thereby enforcing immune‐synapse formation and redirecting cytotoxic activity toward malignant cells [139]. To mitigate class‐typical cytokine‐release syndrome (CRS) and immune‐effector cell–associated neurotoxicity syndrome (ICANS), contemporary programs commonly employ half‐life–extended IgG‐like formats together with step‐up dosing [140]. In lung cancer, the DLL3 × CD3 TCE tarlatamab has been at the forefront of development [141]. Most SCLC display high levels of DLL3 on the tumour cell surface, consistent with DLL3's function as an inhibitory Notch ligand associated with neuroendocrine differentiation [142]. Phase 1/2 studies in previously treated SCLC demonstrated durable responses with manageable safety [143], which supported the U.S. FDA's accelerated approval in May 2024 for extensive‐stage SCLC after platinum chemotherapy. The subsequent phase 3 DeLLphi‐304 trial showed a significant OS advantage over chemotherapy, with most CRS and ICANS events being low grade and manageable under step‐up dosing [141]. As of now, no TCE has been approved for NSCLC. Additional DLL3/CD3 agents and other solid‐tumour TCEs are in early‐ to mid‐phase development, and successful application beyond neuroendocrine histologies will likely depend on target selection, exposure optimization, and toxicity management [139].

### 11.9 Adoptive T‐Cell Therapies: TIL, TCR‐T, and CAR‐T

Adoptive T‐cell therapies can be grouped into three modalities: TIL therapy (expansion of tumour‐infiltrating lymphocytes), TCR‐T cells (genetic transfer of HLA‐restricted endogenous TCRs), and CAR‐T cells (HLA‐independent recognition of surface antigens via an antibody‐derived single‐chain variable fragment). In lung cancer, the TIL product lifileucel has shown Phase II signals of efficacy and tolerability in previously treated NSCLC [144]. For TCR‐T therapy, a MAGE‐A4–targeted product has reached regulatory implementation in synovial sarcoma on Phase II data [145], indicating platform maturity although not approved for lung cancer; in NSCLC, programs targeting endogenous antigens such as KRAS mutations are in development [146]. For CAR‐T cells, ROR1‐targeted constructs have demonstrated generally acceptable safety in Phase I, while antitumor activity in epithelial malignancies, including NSCLC, has been limited [147]. By contrast, an IL‐18–secreting “armoured” CAR‐T targeting DLL3 has shown strong activity in preclinical SCLC models and is moving into early clinical testing [148]. Overall, capitalising on the multi‐antigen breadth of TILs, the precision of TCR‐T cells, and the HLA‐independent targeting of CAR‐T cells, together with rational combinations such as PD‐(L)1 blockade, metabolic or cytokine support, and locoregional delivery, will be central to overcoming TME constraints.

## 12 Conclusion

T cells lie at the core of antitumor immunity in lung cancer, and their functional status critically determines both prognosis and therapeutic outcomes. While therapies targeting immune checkpoint molecules have already demonstrated significant clinical benefit, challenges remain in terms of limited response rates and the emergence of resistance. Overcoming these hurdles and developing novel therapeutic strategies will require a deeper understanding of the immune dynamics within the TME, particularly those involving T cells.

## Funding

This research was supported by the Japan Society for the Promotion of Science (JSPS) (JP24K02549 [Y.T.]); the Japan Agency for Medical Research and Development (AMED) (Practical Research for Innovative Cancer Control, JP25ck0106001h0001 [Y.T.]; Project for Promotion of Cancer Research and Therapeutic Evolution, JP23ama221325h0001 [Y.T.]); the Japan Science and Technology Agency (JST) (FOREST, JPMJFR2049 [Y.T.]).

## Conflicts of Interest

Yosuke Togashi received honoraria from Ono Pharmaceutical, Bristol‐Myers Squibb, Chugai Pharmaceutical, AstraZeneca, Eisai, and MSD; and research grants from Daiichi‐Sankyo, Janssen Pharmaceutical, AstraZeneca, KORTUC, Takeda, and Taiho outside this study. All the other authors declare no conflicts of interest.
